# Supplementary material for: Trend and epidemiological patterns of animal bites in Golestan province (Northern Iran) between 2017 and 2020
Source: PLoS One. 2021 May 25;16(5):e0252058. doi: 10.1371/journal.pone.0252058 (PMC8148354; doi:10.1371/journal.pone.0252058)
Supplement: Attachment — Submitted filename: Response to Reviewers.docx [file pone.0252058.s002.docx]

| *Dear Editor and Reviewers*  *We made the requested corrections*  *- Details of the corrections are listed in the table below.*  *- Referees' corrections are marked in highlighted form. (In Revised Manuscript with Track Changes file)*  *- Professional text editor corrections are also marked as review markup (In Revised Manuscript with Track Changes file)*  *- The letter of the professional text editor certificate is also attached.* | | | | | |
| --- | --- | --- | --- | --- | --- |
| Action taken | **comment** | **Recommender** | **section** |  |  |
| corrected | Please ensure that your manuscript meets PLOS ONE's style requirements, including those for file naming… | Editor | General |  |  |
| - corrected( (source defined))  - In our study, all ethical standards and the principle of confidentiality of information have been observed and approved by the ethics committee, which is mentioned in the method section.  -This study was not prospective and the authors had access to anonymous patient information. | Please clarify the source of the socio-demographic data that were used for this study. If prospectively obtained, please clarify the consent process that was used to collect the data…. | Editor | Method |  |  |
| corrected | In the Methods section please provide the source of the national and provincial data | Editor | Method |  |  |
| corrected  (The manuscript editing was done by a professional service. An editing certificate was attached) | We suggest you thoroughly copyedit your manuscript for language usage, spelling, and grammar. If you do not know anyone who can help you do this, you may wish to consider employing a professional scientific editing service. | Editor | General |  |  |
| ORCID id was registered. | PLOS requires an ORCID ID for the corresponding author in Editorial Manager on papers submitted after December 6th, 2016 | Editor | General |  |  |
| We changed the software used in the design of Figure 2 (update map) and redesigned it with QGIS software. QGIS is open access software, so publishing this image will not be copyrighted. | We note that Figure 2 in your submission contain map images which may be copyrighted. | Editor | Result  (figure) |  |  |
| corrected  ( page number added) | Please add page and line numbers to the document | R1 | General |  |  |
| corrected | Please indent the first line of each paragraph. That will make the manuscript easier to follow. | R1 | General |  |  |
| corrected | I think that the manuscript would benefit from the services of an English-language editor. It could be more concise and clear. | R1 | General |  |  |
| corrected | The source data from Golestan and from the entire nation should be described more clearly. | R1 | Method |  |  |
| Considering that the statistical census method was used in this study and the data of all 33,996 victims of animal bites were examined, the results are clear in line with the objectives of the study. | It seems that the Golestan data set might benefit from further analysis. Multiple regression analysis would reveal the strength of associations between the various demographic and animal features and animal bites. | R1 | Method |  |  |
| corrected | Table 1, I think the factor Nationality can be deleted. | R1 | Result  (Table) |  |  |
| corrected | The title of Table 2 could be better: “Temporal aspects of the animal bites.” | R1 | Result  (Table) |  |  |
| corrected | Tables 2 and 3 have been switched. The text has Table 2 as the type of biting animal, but that information is shown in current Table 3. | R1 | Result  (Table) |  |  |
| corrected | In Table 3, the data on number and percent are switched from their proper headings. | R1 | Result  (Table) |  |  |
| corrected (Explained more) -  -In our study, the incidence rates, especially ascending or descending, are important because they indicate the success or failure of animal bite and rabies control programs in that geography. | Figures 1 and 2 are shown, but you say little about them. Why are they important? Can you elaborate a bit? | R1 | Result |  |  |
| Corrected-  - Rabies prevention services are equally available throughout the province (in 22 boarding centers).  - About the outcomes of treatment was added to the text (in result and discussion)  - While confirming the opinion of the esteemed referee, how to improve the performance of the treatment services is not one of the objectives of the study. | After Table 2, there is text on the completeness of treatment at the clinics. This seems extremely important to me. What is known about the outcomes of treatment? What can be done to improve the performance of the treatment services? Is the rabies service equally accessible in the different parts of Golestan? You do not show data on these points, and you do not discuss services. | R1 | Result |  |  |
| - corrected ( Was summarized ) | I would delete the first sentence. You stated this in the Introduction | R1 | Discussion |  |  |
| corrected | The final 2 words in the Discussion are out of place | R1 | Discussion |  |  |
| - YES  [(Epidemiology – WHO: Cross-sectional studies are a relatively quick way of getting an estimate of disease incidence or prevalence in a community. …](http://( Epidemiology – WHO: Cross-sectional studies are a relatively quick way of getting an estimate of disease incidence or prevalence in a community. …www.who.int › water_sanitation_health › publications))  [www.who.int › water_sanitation_health › publications)](http://( Epidemiology – WHO: Cross-sectional studies are a relatively quick way of getting an estimate of disease incidence or prevalence in a community. …www.who.int › water_sanitation_health › publications)) | Let me know that in cross sectional study design, can we calculate incidence? | R2 | Method |  |  |
| corrected | In the method you mentioned that you used descriptive study such as mean and SD but I could not see it in the results | R2 | Method |  |  |
| corrected | In the table there are some values like 88/9, 1/1 which is not understandable. | R2 | Result  (Table) |  |  |
| corrected | It also need English editing | R2 | General |  |  |
