# Supplementary material for: Trend and epidemiological patterns of animal bites in Golestan province (Northern Iran) between 2017 and 2020
Source: PLoS One. 2021 May 25;16(5):e0252058. doi: 10.1371/journal.pone.0252058 (PMC8148354; doi:10.1371/journal.pone.0252058)
Supplement: Attachment — Submitted filename: Response to Reviewers.docx [file pone.0252058.s003.docx]

| *Dear Editor and Reviewers*  *We made the requested corrections*  *- Details of the corrections are listed in the table below.*  *- Referees' corrections are marked in highlighted form. (In Revised Manuscript)*  *- The letter of the professional text editor certificate is also attached.* | | | | | |
| --- | --- | --- | --- | --- | --- |
| Action taken | **comment** | **Recommender** | **section** |  |  |
| There was no retracted article in our reference | Please review your reference list to ensure that it is complete and correct. If you have cited papers that have been retracted, please include the rationale for doing so in the manuscript text, or remove these references and replace them with relevant current references. Any changes to the reference list should be mentioned in the rebuttal letter that accompanies your revised manuscript | Editor | Reference |  |  |
| - Corrected | I would like to suggest some further edits for clarity and concision.  "Background: Rabies is one of the oldest zoonosis viral diseases… Methods: This cross-sectional study examined epidemiologic …" | R1 | Abstract |  |  |
| - Corrected | -I would re-write the first sentence of the second paragraph thus: The virus is distributed worldwide with the exception of Antarctica. Then the next sentence can be deleted. | R1 | Introduction |  |  |
| - Deleted | .- I think the first sentence of the final paragraph of the Introduction can be deleted. | R1 | Introduction |  |  |
| - Corrected | I would shorten the first paragraph:  "We conducted a cross-sectional study…" | R1 | Method |  |  |
| - Corrected | I would shift one sentence to make this the first sentence of the second paragraph: Golestan provincial data reflecting epidemiological … | R1 | Method |  |  |
| - Corrected | I would re-write the sentence that currently begins with “As well, national data...” thus: National data on overall and province-specific incidence rates 2013-2020 were collected from the Center for Communicable Diseases Control.. | R1 | Method |  |  |
| - Corrected | In the first paragraph, I would re-write the sentence that currently begins “Of note…  In Golestan province, Gomishan, Aliabad,…." | R1 | Result |  |  |
| - Corrected | I would make the names of the Figures more specific: Fig.1. Incidence trend of animal bites per 100,000 people in Golestan province and in Iran Fig. 2. Animal bite incidence rates in Golestan counties (2013-2020) | R1 | Result |  |  |
| - Corrected | In the paragraph that describes the data in Table 1, if you add (Table 1) to an earlier sentence, you can delete the last sentence that currently reads “Some demographic information of the patients are shown in Table 1. That sentence does not really add much to the text. | R1 | Result |  |  |
| - Corrected | In the text on Table 2, and in the Table itself, I think the hour intervals should be 0-6, 6-12, 12-18, and 18-24. | R1 | Result |  |  |
| - Corrected | In the paragraph discussing Table 3, I think you should move the first two sentences should be moved to the end of the paragraph. That way, the structure of the text and of the Table are the same. | R1 | Result |  |  |
| - Corrected | I would re-structure the first paragraph thus: "According to the World Health Organization’s report, Asia …" | R1 | Discussion |  |  |
| - Corrected | I would re-write some of the next paragraph: " Our data showed that the most animal bites in Golestan occurred in men…" | R1 | Discussion |  |  |
| Iran's Health System follows the protocols of the World Health Organization in providing post-exposure prophylaxis (PEP) services and it can be said with certainty that the report of one case of human rabies in the geography of our study, which has a high rate of animal bites, indicates the appropriate coverage of PEP services.  On the other hand, a high percentage of incomplete vaccine course or a low percentage of immunoglobulin injection alone, do not indicate a deficiency of prevention service, because according to treatment protocols, if the invading animal survives after ten days, there is no need to complete the course of vaccine . Also according to the World Health Organization protocol immunoglobulin is injected only to the third type of exposure. | I hope you can explain what you mean in the final sentence above; it is unclear to me.  Near the end of the Discussion, bottom of page 7, you write that only one case of human rabies resulted from this very large number of bites. You speculate that this likely resulted from the rather complete coverage of POST-exposure prophylaxis. But your own data in Table 3 shows that the great majority of cases had incomplete vaccine provided, and few received immunoglobulin. It seems very inconsistent to me. Perhaps all pet dogs and cats are vaccinated, thereby resulting in PRE-exposure prophylaxis? This is my big question, and I hope you can clarify it for me and other readers | R1 | Discussion |  |  |
| .  -Yes, cross-sectional and cohort studies are suitable for calculating the incidence and prevalence, respectively, but according to some sources to reduce the problems of studies and the nature of some variables ( Like animal bites, which due to their non-chronic nature, there is no difference between its prevalence and incidence ), incidence can be calculated by cross-sectional study. Two sources have been attached for the visit.  -Also, if the reviewer is not satisfied with the quality of the attached references, we can replace the retrospective cohort study with a descriptive approach (or…), because the variable nature of the animal bite and the quality of our data (collected chronologically) allow us to do so, there is no change in the results.  1. Page 4, last paragraph:  <https://www.who.int/water_sanitation_health/economic/>  chapter4.pdf  [(Epidemiology – WHO: Cross-sectional studies are a relatively quick way of getting an estimate of disease incidence or prevalence in a community. Cross-sectional studies look at the disease status of all or a sample of a population at a particular moment in time. In general each individual would be contacted only once. For diseases with seasonal variation in their incidence the results of the survey would clearly depend on what time of year the study was carried out.](http://( Epidemiology – WHO: Cross-sectional studies are a relatively quick way of getting an estimate of disease incidence or prevalence in a community. …www.who.int › water_sanitation_health › publications))  2. Pharmacoepidemiology in the Prevention of Adverse Drug Reactions (Chapter 3 , 3.4.2.4 The Cross-Sectional Study)  <https://www.sciencedirect.com/topics/nursing-and-health-professions/cross-sectional-study>  The incidence prevalence, important descriptive measures, can also be calculated from a cross-sectional study | I could not find anything in your reference about calculation of incidence in cross sectional study. In my point of view we cannot calculate incidence in cross sectional study but we can calculate prevalence. For calculation of incidence we have to conduct cohort study. | R2 | Method |  |  |
