# Supplementary material for: Trend and epidemiological patterns of animal bites in Golestan province (Northern Iran) between 2017 and 2020
Source: PLoS One. 2021 May 25;16(5):e0252058. doi: 10.1371/journal.pone.0252058 (PMC8148354; doi:10.1371/journal.pone.0252058)
Supplement: Attachment — Submitted filename: Response to Reviewers.docx [file pone.0252058.s004.docx]

| *Dear Editor and Reviewers*  *We made the requested corrections*  *- Details of the corrections are listed in the table below.*  *- Referees' corrections are marked in highlighted form. (In Revised Manuscript)*  *- The letter of the professional text editor certificate is also attached.* | | | | | |
| --- | --- | --- | --- | --- | --- |
| Action taken | **comment** | **Recommender** | **section** |  |  |
| - Corrected | Please include your tables as part of your main manuscript and remove the individual files. Please note that supplementary tables (should remain/ be uploaded) as separate "Supporting Information" files | Editor | Result  -Table |  |  |
| We received this comment in the first round of reviewing of article and corrected it as follows:  We changed the software used in the design of Figure 2 and our team redesigned it with QGIS software. QGIS is open access software, so publishing this image will not be copyrighted. | We note that Figure 2 in your submission contains a map which may be copyrighted. .. | Editor | Result  -Figure |  |  |
| - Corrected | In Table 2, it looks like the lowest bite rate was from midnight to 6AM (3%). I think you need to revise that sentence at the bottom of page 4. | R1 | Result |  |  |
| - Corrected (In Table 1) | In Table 2, can you change the label from ‘free’ to ‘freelancer’? That way, it will match the text in the Discussion.    . | R1 | Result |  |  |
| - Corrected | . At the top of page 5, the sentence could be made clearer if you write: “...96% of total bites; 49% of these animals had owners.” | R1 | Result |  |  |
| - Thanks to the point of view of Honorable reviewer, paragraph 7 refers to the coverage of Pep services, which includes the provision of standard services. | In the middle of page 7 where you discuss the extraordinarily low death rate from rabies, the text appears inconsistent with the data on completeness of treatment. But the explanation you kindly provided in your response to reviewers taught me why there is no real inconsistency. (I am not a rabies expert!) I suggest that matters would be clarified in the article if you add some of the text from your response to reviewers here in the Discussion. | R1 | Discussion |  |  |
